# MYB30 regulates root cell elongation under abscisic acid signaling

**DOI:** 10.1080/19420889.2018.1526604

**Published:** 2018-10-12

**Authors:** Satomi Sakaoka, Kaho Mabuchi, Atsushi Morikami, Hironaka Tsukagoshi

**Affiliations:** a Faculty of Agriculture, Meijo University, Nagoya, Aichi, Japan; b Graduate School of Bioagricultural Science, Nagoya University, Nagoya, Aichi, Japan

**Keywords:** Abscisic acid, plant hormone signaling, MYB30, reactive oxygen species, root cell elongation, crosstalk

## Abstract

Reactive oxygen species (ROS) and plant hormones play important roles in regulating plant growth and stress responses as signaling molecules. Abscisic acid (ABA) is known as the key regulator of both abiotic and biotic stress responses. During stress responses, ABA is known to regulate ROS production, indicating that important crosstalk occurs between ROS and ABA signaling. We recently reported that MYB30, an MYB-type transcription factor, regulates root cell elongation under ROS signaling. In this study, we analyzed the molecular interaction between ROS and ABA signal during for root development, which is mediated through MYB30 transcriptional regulation. We showed that MYB30-regulated root cell elongation was mediated by ROS production under ABA signaling. Our findings will provide one piece of evidence of the complex cross talk between ROS and hormone signaling that regulates root development.

Since plants grow where they germinate, understanding their growth cycle is important in dealing with surrounding environmental changes and crucial for ensuring plant survival. Furthermore, plant roots play an important role in sensing environmental changes in the soil and adapting the entire plant body to these changes. The molecular mechanisms that regulate plant growth responses to environmental changes have been well studied using model plants such as *Arabidopsis thaliana* [1]. Many of these studies reported that plant hormones play a key role in plant growth regulation. Furthermore, recent studies have revealed that reactive oxygen species (ROS) also have an important function in regulating plant growth [2]. In fact, several studies have reported that the crosstalk between plant hormones and ROS signaling is also an important regulatory mechanism for plant growth [3].

Maintaining the balance between cell proliferation and differentiation is a key event in ensuring normal root growth. Plant hormones such as auxin and cytokinin act as key signaling molecules for regulating this balance. Under auxin signaling, PLETHORAs (PLTs) are known as key regulators of the maintenance of QC nitch cell activity at the root tip [4,5], and the balance between cell proliferation and differentiation. Auxin and cytokinin have been reported to create a transcriptional circuit for regulating PLT activity [6]. In addition to auxin and cytokinin, Jasmonate is known as a regulator of *PLT* expression through MYC2 [7]. Root growth factor (RGF), which is a peptide hormone, also regulates PLT activity [8]. These findings indicate that regulation of cell proliferation by PLT is one of the major events in the root meristematic zone for controlling root growth. However, there are several different regulations of the meristematic activity from PLT. One example is the transcriptomic regulation by UP BEAT1 (UPB1), which regulates ROS homeostasis at the root tip, and this homeostasis regulates the balance between cell proliferation and differentiation [9]. Hydrogen peroxide (H_2_O_2_) treatment reduces root growth, which shortens both meristem size and cell length. In this case, *PLT* expression does not change much but the expression of cell cycle-related genes is downregulated in H_2_O_2_-treated roots [10]. These reports indicate that ROS signaling might be mediated act via different pathways from those regulated by PTL. However, ABA, which is known as a hormone that regulates plant responses to abiotic stress [11], influences PLT protein level and root growth [12] through the production of ROS in the mitochondria of the root tip. All these studies suggest that complex crosstalk between plant hormones and ROS signaling is involved in regulating root growth.

We recently reported that a MYB type transcription factor, MYB30, controls root cell elongation under ROS signal in *A. thaliana* [13]. MYB30 is upregulated by ROS, and *MYB30* target genes reduce cell elongation. During MYB30 function analysis, we also discovered that MYB30 signaling seems to be independent of that of auxin and cytokinin. However, further analyses are still required to elucidate the relationship between ROS and plant hormones. Especially, ABA is known as a regulator of ROS production under abiotic stresses [12,14,15]. In this study, we analyzed the relationship between *MYB30* gene regulatory network and plant hormones such as auxin, cytokinin, and ABA. Our findings will provide one piece of evidence supporting the existence of complex crosstalk between ROS and hormone signaling that regulates root development.

## Roots of *myb30* mutants were longer than those of wild-type following ABA treatment

**Figure 1.**
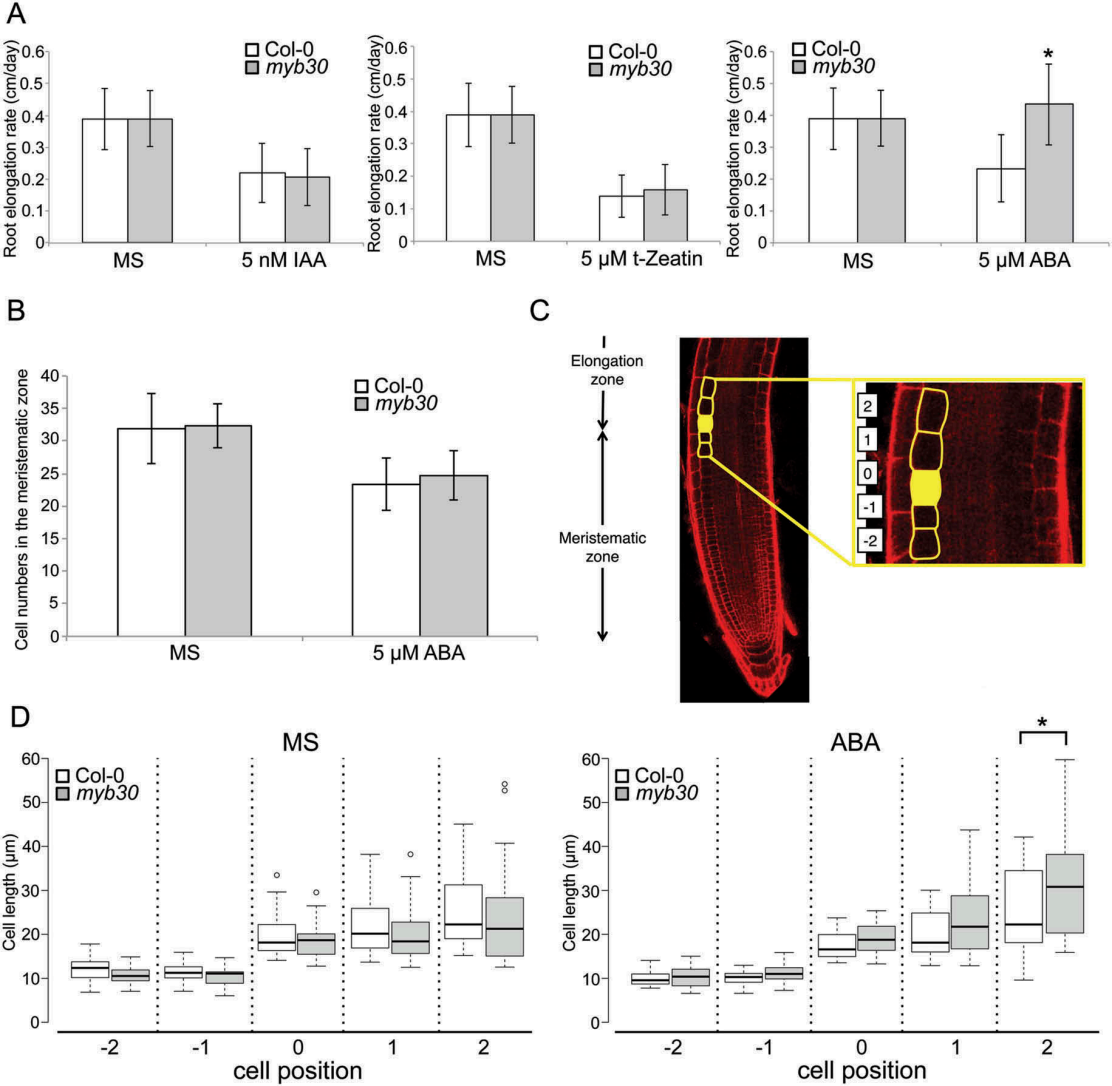
Root phenotypes of *myb30* mutant treated with abscisic acid (ABA). (a) Average root elongation rate (cm/day) of Col-0 (white boxes) and *myb30* (grey boxes) that were treated with control, 5 nM indole-3-acetic acid (IAA), 5 µM t-zeatin, and 5 µM ABA (n = 30, means ± standard deviation [SD]); *p < 0.05, determined using Student’s *t*-test. (b) Cortex cell numbers in meristematic zone of 5 dai plants of Col-0 (white boxes) and *myb30* mutant (gray boxes) treated with control Murashige and Skoog (MS) medium and 5 µM ABA for 1 day (n = 22, means ± SD). (c) Positions of cells for measurement of cell length at boundary between meristematic and elongation zone. Position “0” (first cell in the elongation zone) was defined as cells that were 1.5 times longer than one cell below, and the cell length was more than 15 µm (yellow-filled). Cells adjacent one above the other to position 0 were numbered as position “1” and “-1” (yellow frames). Adjacent cells of position “1” and “-1” were numbered as positions “2” and “-2,” respectively (yellow frames). (d) Cell length of Col-0 (white boxes) and *myb30* (gray boxes) exposed to control MS and 5 µM ABA treatment for 1 day (n = 22); *p < 0.05, determined using Student’s *t*-test.

ROS accumulation is known to be associated with endogenous ABA levels in plant cells, so we hypothesized that the ROS signal would be related with ABA during root development. Since we previously demonstrated that MYB30 plays a role as a regulator of root cell elongation following H_2_O_2_ treatment, we examined the role of MYB30 in the effects of ABA treatment. When we treated *myb30* mutants with 5 µM ABA for 24 h (Figure 1(a)), they showed longer roots than the *A. thaliana* Columbia-0 (Col-0) wild-type did. We also compared the effects of other hormones such as auxin and cytokinin on root elongation to those of *myb30* (Figure 1(a)). The root elongation rates of Col-0 and *myb30* were comparable following both auxin and cytokinin treatments.

We then counted cell numbers in the meristematic zone of Col-0 and *myb30* exposed to control and 5 µM ABA treatments (Figure 1(b)). Cell numbers in the meristematic zone were comparable in both the control and ABA-treated roots of Col-0 and *myb30* plants. We also measured the cell length at the boundary between the meristematic and elongation zone (Figure 1(c, d)). The result showed that *myb30* plants exhibited slightly but significantly longer cells in the elongation zone than Col-0 plants did after ABA treatment. However, the cell size in the meristematic zone was not different between Col-0 and *myb30* even after ABA treatment. These results indicate that MYB30 also has a regulatory function in root cell elongation following ABA treatment.

## *MYB30* was upregulated by ABA treatment

**Figure 2.**
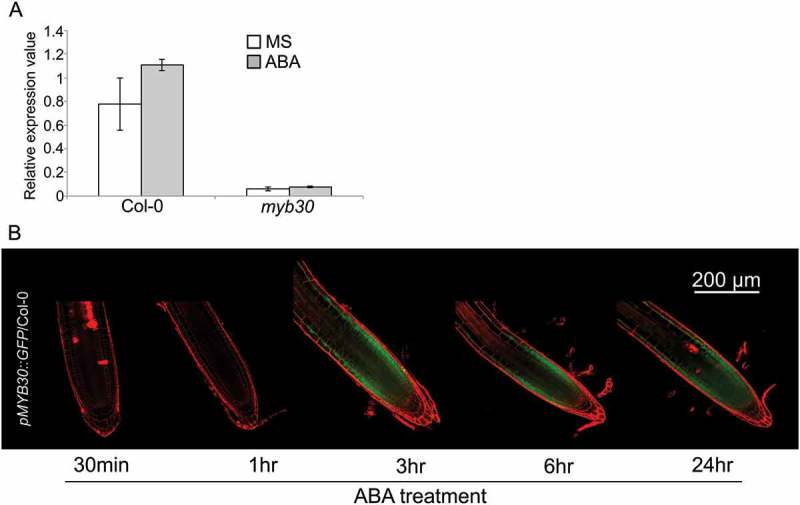
*MYB30* expression patterns after abscisic acid (ABA) treatment. (a) *MYB30* expression in 5 days after imbibition (dai) Col-0 and *myb30* root meristem zones treated with control Murashige and Skoog (MS) medium (white boxes) and 5 µM ABA (gray boxes)for 1 day (n = 3, means ± standard deviation [SD]). (b) Expression of 5 dai *pMYB30::GFP* in Col-0 plants (transcriptional fusion) treated with 5 µM ABA for 0, 1, 3, 6, and 24 h. Scale bar, 200 µm.

We also examined MYB30 expression levels using reverse transcription-quantitative polymerase chain reaction (RT-qPCR) and the *MYB30* transcriptional fusion line (*pMYB30::GFP*) to determine if ABA could regulate *MYB30* expression. In Col-0 wild-type but not *myb30* plants, *MYB30* was upregulated by ABA treatment in the RT-qPCR analysis (Figure 2(a)). However, the induction level of *MYB30* by ABA was weaker than that by H_2_O_2_ treatment when we compared *MYB30* expression level with that of our previous dataset [13].

In the transcriptional fusion assay, we treated *pMYB30::GFP* in the Col-0 line with 5 µM ABA for 1, 3, 6, and 24 h (Figure 2(b)). The green fluorescence protein (GFP) fluorescence was intensified in all meristematic zones and epidermal cells in the elongation zone after 3 h treatment. Furthermore, GFP level was not changed after 24 h treatment with ABA, and these results indicate that *MYB30* is an ABA-responsive gene.

## ROS levels were not changed in *myb30* after ABA treatment

**Figure 3.**
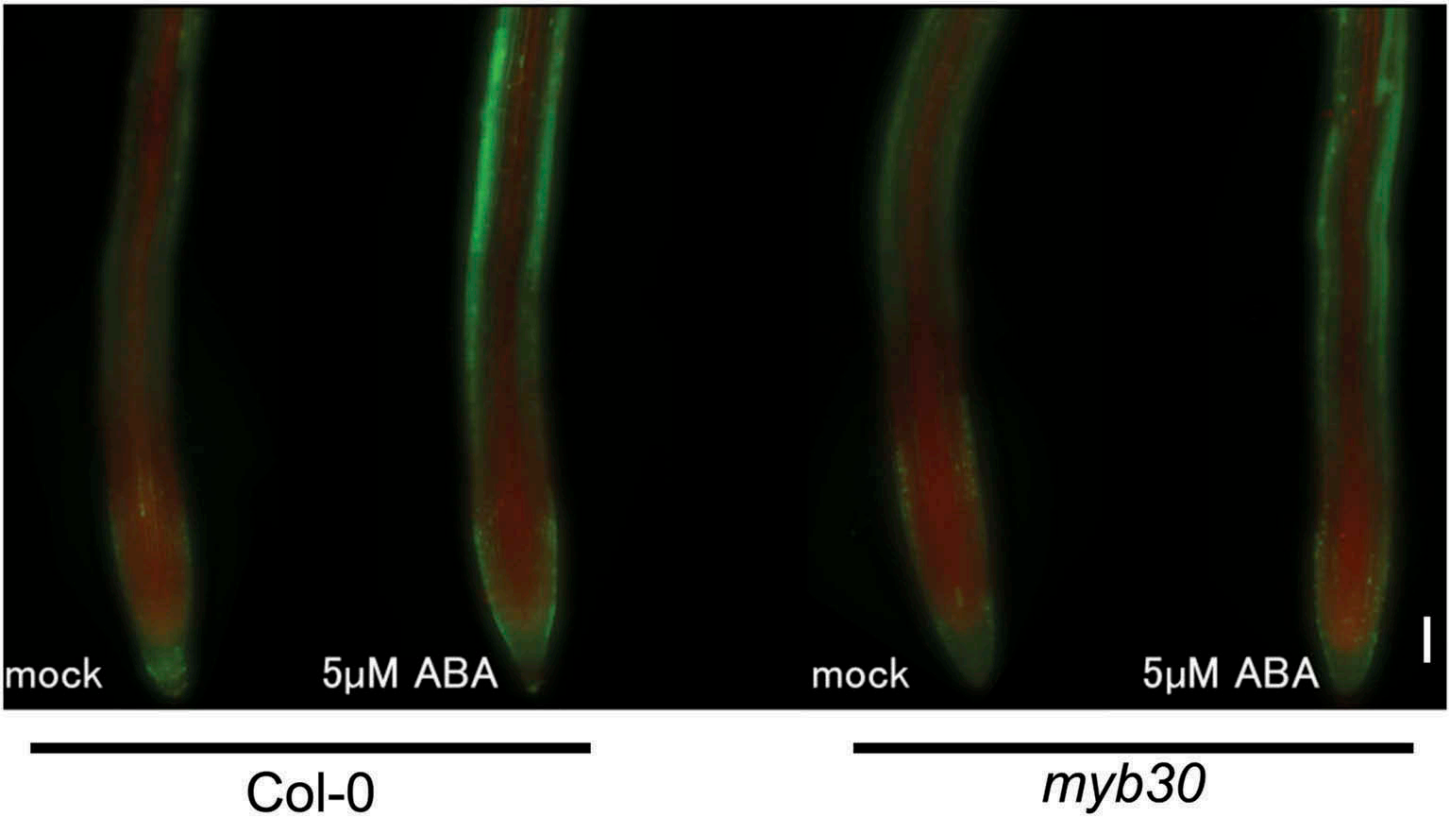
Reactive oxygen species (ROS) distribution in *Arabidopsis thaliana* Columbia-0 (Col-0) and *myb30* mutant roots. The 5 days after imbibition (dai) Col-0 and *myb30* mutant root meristem zones treated with control Murashige and Skoog (MS) medium and 5 µM ABA for 1 day and stained with both dihydroethidium (DHE) and BES- H_2_O_2_-Ac. Red and green fluorescence, DHE and BES- H_2_O_2_-Ac signals, respectively. Scale bar, 100 µm.

In guard cells and plant shoot parts, ROS levels have been reported to be increased by ABA [16]. To investigate whether ABA induces ROS levels in the root, we stained Col-0 and *myb30* specimens with dihydroethidium (DHE) and BES-H_2_O_2_-Ac (O_2_^−^ and H_2_O_2_ indicators, respectively) after 24 h treatment with 5 µM ABA (Figure 3). DHE fluorescent was strong in the meristematic zone than in other parts, and BES-H_2_O_2_-Ac fluorescence was detected mainly in the elongation zone in both Col-0 and *myb30* specimens. The intensity of both ROS indicators became stronger following ABA treatment in both Col-0 and *myb30*. These results indicate that *MYB30* mutation did not affect the alteration of ROS biosynthesis under ABA treatment, which was most likely due to the lack of an *MYB30* gene regulatory network after ROS production by ABA.

## Myb30-regulated genes were involved in controlling root cell elongation

**Figure 4.**
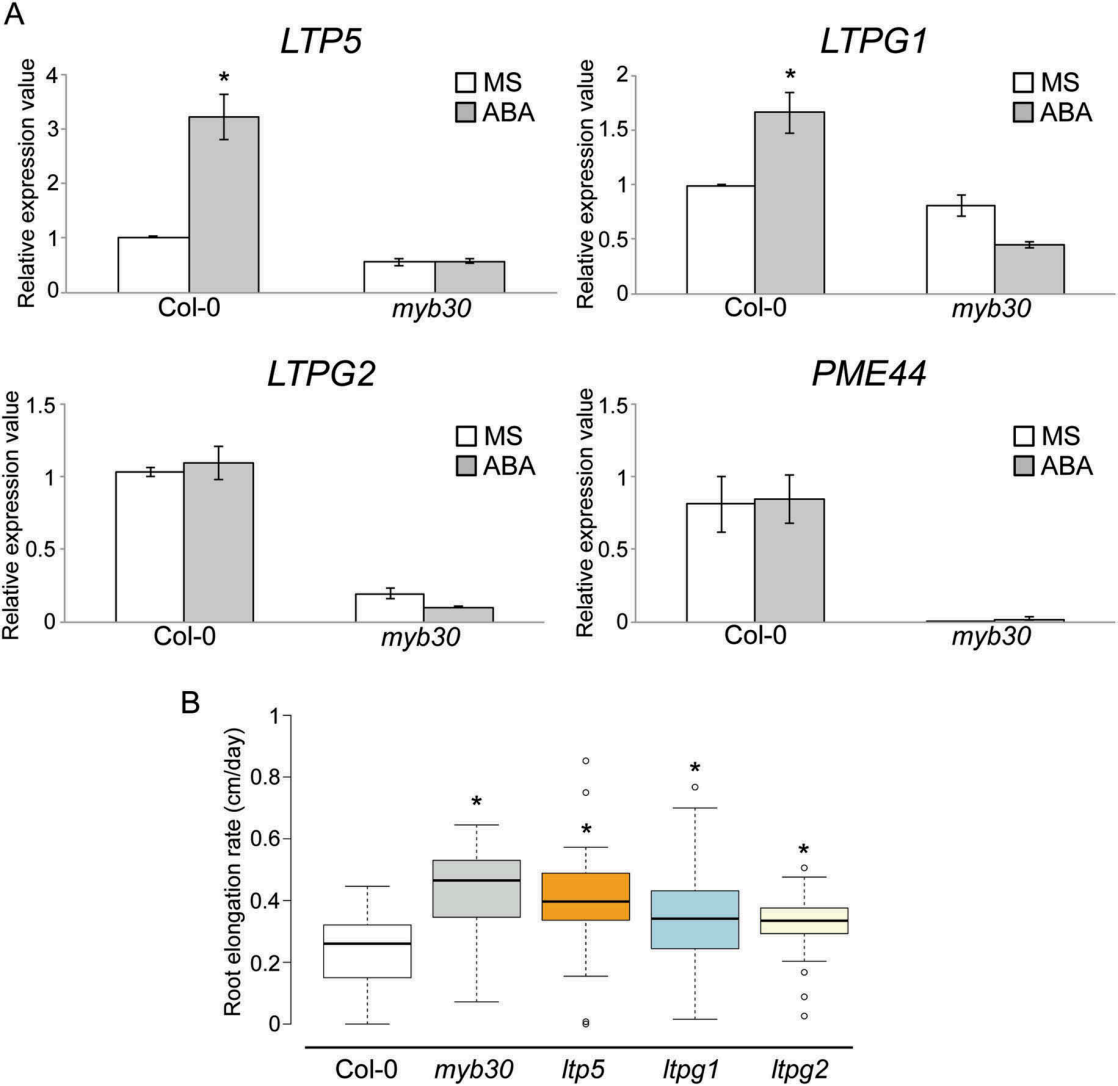
Expression patterns of MYB30 direct target genes and root phenotype of abscisic acid (ABA)-treated mutants. (a) Reverse transcription-quantitative polymerase chain reaction (RT-qPCR) analysis of expression of MYB30 target genes in 5 dai root tip of *Arabidopsis thaliana* Columbia-0 (Col-0) and *myb30* treated with control Murashige and Skoog (MS) medium (white boxes) and 5 µM ABA (gray boxes) for 1 day (n = 3, means ± standard deviation [SD]); *p < 0.01, determined using Student’s *t*-test. (b) Average root elongation rate (cm/day) of Col-0 (white box), *myb30* (white box), *ltp5* (orange box), *ltpg1* (light blue box), and *ltpg2* (light yellow box) treated with 5 µM ABA for 1 day (n = 30, means ± SD); *p < 0.01, determined using Student’s *t*-test compared to Col-0.

We previously reported on four MYB30 direct target genes, lipid transfer protein 5 (*LTP5), LTPG1, LTPG2*, and pectin methylesterase 44 (*PME44*) [13]. We examined changes in the expression levels of these four genes in Col-0 and *myb30* roots exposed to control and 5 µM ABA treatments for 24 h using RT-qPCR (Figure 4(a)). All four genes were strongly downregulated in *myb30* roots, which was consistent with our previous results [13]. Furthermore, *LTP5* and *LTPG1* but not *LTPG2* and *PME44* were significantly upregulated by ABA treatment in Col-0 roots.

We then determined if Col-0, *myb30, ltp5, ltpg1*, and *ltpg2* mutants exhibited a significant difference in root elongation following 24 h ABA treatment (Figure 4(b)). Similar to the expression analysis, *ltp5* and *ltpg1* mutants showed higher elongation rate than Col-0 did after ABA treatment. These results suggest that LTP5 and LTPG1 likely have a primary role in regulating root elongation following ABA treatment in the *MYB30* gene regulatory network.

## Interaction between MYB30 and plant hormones in root growth

We previously reported that MYB30 has important functions as a root growth regulator under ROS signaling [13]. During the analysis of MYB30 function, we also found that it seems to act independently of plant hormone signals such as auxin and cytokinin. To confirm this finding, we investigated the effects of auxin and cytokinin on the root growth of *myb30*. The results of the root growth analysis showed that auxin and cytokinin had similar effects in reducing the root growth of both Col-0 and *myb30*. However, another plant hormone, ABA, induced *MYB30* expression in the root. Not only *MYB30* expression, but *myb30* showed significant insensitivity to the root growth inhibition after ABA treatment. These results indicate that MYB30 most likely acts independent of auxin and cytokinin in root growth but MYB30 plays some regulatory roles in root growth under ABAs signaling. ABA is known to induce ROS cellular level in guard cells and the mitochondria at the root tip [12,15]. In addition, MYB30 is reported to be involved in seed germination under ABA signaling [17]. Moreover, MYB30 protein levels are stabilized by a SUMO E3 ligase, SIZ1, and this stabilization is important in regulating the expression of certain ABA response genes [18]. According to these previous findings and our results, MYB30 might function as a root growth regulator under ABA signaling and stress responses.

## MYB30 functions under ABA signal as a root growth regulator

After ABA treatment, Col-0 and *myb30* plants showed similar decreases in root meristem size. Under H_2_O_2_ treatment, the meristem size of *myb30* was the same as that of Col-0 [13]. Similar to H_2_O_2_ treatment, ABA treatment of *myb30* induced longer cell sizes in the elongation zone than the wild-type. These results indicate that MYB30 also regulated cell elongation not the cell proliferation under both ABA and ROS signals. This also indicated that MYB30 regulates root cell elongation through the same MYB30 gene regulatory network both in ABA and ROS.

After ABA treatment, *MYB30* was up-regulated, but the increase in expression levels was lower than those with H_2_O_2_ treatment. We then hypothesized that *MYB30* induction by ABA treatment is likely indirect. Since ABA induces ROS levels in the root, we stained ROS at the root tip using ROS indicators. ABA induced ROS at the root tip and, so, the low levels of induction of *MYB30* expression by ABA treatment could be upregulated by ROS induced by ABA. In support of this finding, we observed that transcriptional fusion of the *MYB30* promoter showed slightly lower GFP expression after ABA treatment than after H_2_O_2_ treatment. Transcriptional fusion lines exhibited initial weak GFP expression after 1 h treatment with H_2_O_2_ [13], whereas GFP was detected after 3 h treatment with ABA. This time gap also indicates that *MYB30* was regulated by ABA indirectly.

Since *MYB30* mutation could affect the production level of ROS, we also stained *myb30* root tips with ROS indicators, and the results showed that the ROS accumulation patterns were comparable between Col-0 and *myb30* mutants even after ABA treatment. This result strongly suggests that the insensitivity to ABA treatment, which decreased the root elongation rate in *myb30* was mediated by a deficiency in the *MYB30* gene regulatory network that was upregulated by the ROS produced by ABA.

## *MYB30* direct target genes contribute to root growth following ABA treatment

We previously reported that MYB30 regulates root cell elongation by controlling the gene expressions of its direct targets. MYB30 directly regulates *LTP5, LTPG1, LTPG2*, and *PME44* in the roots [13]. We also investigated these gene expression levels after ABA treatment and found that *LTP5* and *LTPG1* were upregulated by ABA treatment. Under H_2_O_2_ treatment, *LTPG2* mainly functioned in root cell elongation [13]; however, under ABA treatment *LTP5* and *LTPG1* exhibited the main functions. To support this observation, we treated *ltp5, ltpg1*, and *ltpg2* mutants with ABA, and they all showed higher elongation rates than the Col-0 wild-type did. Specifically, the *ltp5* mutant showed the highest elongation rate among these three mutants after ABA treatment. This is consistent with the analysis of gene expression levels where *LTP5* showed the highest induction by ABA in the wild-type root. We could not exclude that other ABA-responsive genes that regulate root cell elongation under ABA treatment coregulated root cell elongation with *LTP5*. However, the *MYB30* gene regulatory network obviously plays an important role in root growth regulation in the crosstalk between ROS and ABA signaling.

Recently, it was reported that MYB94 and MYB96, which belong to the same S1 MYB type subgroup of MYB30, regulate gene expression under ABA signaling in plant shoots [19,20]. Under drought stress, *MYB94* and *MYB96* are upregulated, and these MYB transcription factors positively regulate the gene expression involved in very-long chain fatty acid biosynthesis. Then, they control cuticle wax deposition under drought stresses and ABA treatment [20]. However, both *MYB94* and *MYB96* were not expressed in the roots in our previous transcriptome dataset [13]. In addition, in silico analysis also showed that *MYB94* and *MYB96* were not expressed in the root. These results indicate that MYB94 and MYB96 mainly function in the shoot during drought or ABA signaling, whereas MYB30 plays an important role in the root under ABA signaling. This indicates the existence of organ-specific gene regulation among MYB type transcription factors.

In this study, we investigated the function of MYB30 under ABA and ROS signaling. ABA is known as a stress-response plant hormone. MYB30 is one of the intermediate transcription factors that connects ABA signaling and root development through ROS production. At the root tip, ABA accumulates ROS in the mitochondria, and this affects auxin distribution and PLETHORA (PLT), which act as an important auxin-inducible transcriptional regulator of cell proliferation in the meristematic zone [12]. Combining these previous observations with our present findings, ABA could be considered to regulate root growth through different transcription factors that act in a zone-specific manner at the root tip with ROS as the intermediate signal.

## Materials and methods

### Plant materials and growth conditions

Col-0 was used as the wild-type, and the *myb30, ltpg1*, and *ltpg2* mutants were previously described by Mabuchi et al. [13]. The *ltp5* mutant was obtained from SALK collection and the ABRC seed stock center (SALK_039069). This mutant was genotyped using the left-border primer on the T-DNA (LB), a right-side primer on the genome (RP), and a left-side primer on the genome (LP) as follows, LB: 5ʹ-TTTCGCCTGCTGGGGCAAACCAG-3ʹ, RP: 5ʹ-GCCTTGGGTTCTCGACTTAAC-3ʹ, and LP: 5ʹ-GGGTCGATCGACATTTTAATTG-3ʹ.

All seeds were sterilized with 1% bleach and 0.05% Triton X-100 for 5 min and then washed three times with sterilized water. The seeds were germinated on Murashige and Skoog (MS) medium (WAKO) supplemented with 1% sucrose and 1% agarose after 2 days at 4ºC. The plants were grown vertically in a growth chamber (Panasonic) at 22ºC under 16 h light/8 h dark conditions.

For auxin, cytokinin, and ABA treatments, 5 days after imbibition (dai), the seedlings were transferred onto MS medium containing 5 nM indole-3-acetic acid (IAA), 5 µM t-zeatin, and 5 µM ABA, respectively.

### Real-time Rt-qPCR

RNA was isolated from the root tips, which contained the meristematic zone, of micro-dissected 6 dai plants [9] using an RNeasy micro kit (QIAGEN). First strand cDNA was synthesized using ReverTra Ace qPCR RT Master Mix with gDNA remover (TOYOBO). The real-time RT-qPCR was performed using the Thunderbird SYBR qPCR Mix (TOYOBO) using an Illumina ECO system (Illumina). The RT-qPCR efficiency and the cycle threshold (CT) value were determined using the standard curves for each primer set. Efficiency corrected transcript values of three biological replicates of all samples were used for determining the relative expression values. The level of each value was normalized to that of PP2A subunit gene (PDF2; At1g13320) [21]. The primer sets used in this study were previously described by Mabuchi et al. [13].

### Microscopic and phenotypic analyses

To measure the whole root length, roots growing on plates were scanned using a flatbed scanner. The root length was measured on scanned images using the ImageJ software. Laser scanning confocal microscopy was performed using a Leica SP8 system (Leica) on propidium iodide-stained roots. The cell length and cortex cell number were measured from confocal images using the ImageJ software program.

### ROS staining

Single specimens were double-stained with superoxide (O_2_^−^) and H_2_O_2_ by incubating seedlings in 10 µM DHE (Invitrogen) and 50 µM BES-H_2_O_2_-Ac (WAKO) for 30 min in the dark [9]. Images of stained tissue sections were acquired using a Leica SP8 confocal microscope.
